# miR-30d Levels Predict Re-Hospitalization in Patients with Acute Cardiogenic Pulmonary Edema: A Preliminary Study

**DOI:** 10.3390/ijms26031278

**Published:** 2025-02-01

**Authors:** Giordano Bianchi, Barbara Vizio, Ornella Bosco, Martina Schiavello, Paolo Cagna Vallino, Francesca Rumbolo, Fulvio Morello, Giulio Mengozzi, Giuseppe Montrucchio, Enrico Lupia, Emanuele Pivetta

**Affiliations:** 1 Division of Emergency Medicine and High Dependency Unit, Città della Salute e della Scienza di Torino, Molinette Hospital, 10126 Turin, Italy; giordanobia@gmail.com (G.B.); paolo.cagnavallino@unito.it (P.C.V.); fulvio.morello@unito.it (F.M.); enrico.lupia@unito.it (E.L.); 2 Department of Medical Sciences, University of Turin, 10126 Turin, Italy; barbara.vizio@unito.it (B.V.); ornella.bosco@unito.it (O.B.); martina.schiavello@unito.it (M.S.); giulio.mengozzi@unito.it (G.M.); giuseppe.montrucchio@unito.it (G.M.); 3 Residency Programme in Emergency Medicine, University of Turin, 10126 Turin, Italy; 4 Clinical Biochemistry Laboratory, Città della Salute e della Scienza di Torino, Molinette Hospital, 10126 Turin, Italy; francesca.rumbolo@unito.it

**Keywords:** microRNA, acute heart failure, pulmonary edema

## Abstract

Acute cardiogenic pulmonary edema (ACPE) is a common and serious manifestation of heart failure (HF), representing 10–20% of all acute HF admissions. It is associated with elevated in-hospital mortality and high rates of re-hospitalization. MicroRs, like miR-30d, are of particular interest in heart failure due to their regulatory role in gene expression and potential as biomarkers for diagnosing and predicting patient outcomes, especially in high-risk cases such as ACPE. We conducted a cohort study on patients diagnosed with ACPE in the Emergency Department (ED). The circulating levels of miR-30d were analyzed at the time of hospital admission and at one-month follow-up along with other biomarkers. We enrolled 24 ACPE patients and 10 control subjects. Median age was 80.8 years (interquartile range, IQR, 8.2) in ACPE cases, and 78.5 years (IQR 9.8) in controls with a male/female ratio of 2 and 0.66, respectively. In ACPE patients, median cardiac ejection fraction was 42.5%, creatinine 1.63 mg/dL (IQR 1.24), troponin 63.5 ng/dL (58), and NT-proBNP 4243.5 pg/mL (IQR 5846) at ED evaluation. Median concentration of miR30d was 0.81 in controls, and 3.67 and 7.28 in ACPE patients at enrollment time and one month later, respectively. Re-hospitalization occurred in 7 ACPE patients in the following 3 months, and in 9 during the following year. miR-30d had a significant predictive value in assessing the risk of re-hospitalization at both 3 months and 1 year following the initial diagnosis of ACPE, while it did not in assessing the risk of death at 1 year. When compared with the other biomarkers, none of them showed a better accuracy than miR-30d. Our findings suggest that elevated levels of miR-30d are associated with an increased rate of hospital readmission at both 3 months and 1 year after discharge. Larger, multicenter studies will be needed to confirm the validity of circulating miR-30d levels as a potential biomarker useful for risk prediction in ACPE patients and its utility in improving individualized patient care.

## 1. Introduction

Heart failure (HF) is a complex syndrome characterized by the heart’s inability to pump blood efficiently to meet the body’s metabolic demands [1]. It can occur due to various underlying diseases, including vascular, infective, and autoimmune diseases as well as cardiomyopathies. The prevalence of HF (1–2% of adults) increases with age (around 10% in individuals over the age of 70) and remains somehow underestimated because of a quota of unrecognized cases [1].

Acute heart failure (AHF) is a precipitating clinical condition with multiple causes (coronary artery disease, hypertension, and valve disease represent predominant but not exclusive factors in western countries) that may be, more frequently, an acute manifestation of decompensated HF or the first manifestation of new onset disease that evolves to a chronic condition (CHF). Acute cardiogenic pulmonary edema (ACPE) constitutes 10–20% of all AHF cases and presents as a severe clinical manifestation of left-sided HF. [1].

ACPE patients most frequently are admitted to the emergency department (ED) with severe dyspnea, hypoxia, and lower limb swelling [2]. The diagnosis of ACPE is usually established, after evaluating symptoms and clinical signs, using a consolidated diagnostic work-up, which includes electrocardiogram (ECG), pulse oximetry, chest X-ray, lung ultrasound (LUS), echocardiography, and a panel of laboratory tests (e.g., troponin and NT-proBNP among others) [1,3,4]. ACPE is associated with a high in-hospital death rate (up to 10–20% of cases), and, in those patients who survive the acute phase, with a high re-hospitalization rate and one-year mortality (up to 30%) [5,6,7].

microRNAs (miRs) are small non-coding RNAs that play relevant roles in regulating gene expression and in intercellular signaling [8,9]. In recent years, much progress has been made in elucidating the origin and functions of miRs and their potential use in research and clinical practice [10,11,12].

Also, in the context of HF, several studies have suggested that miRs may have a role both as mediators of disease development and progression and as diagnostic and/or prognostic biomarkers [13,14,15,16]. Among the many miRs investigated, miR-30d has emerged as one of the most promising. miR-30d is a member of a family of almost identical miRs (miR-30a, b, c, d, and e) that are abundant and present at similar levels in normal hearts of humans and mice [17,18]. Among these, miR-30d is the most abundant miR-30 family member in plasma and its expression is downregulated by approximately 20% in the myocardium of mice early after experimental pressure overload induced by surgical transverse aortic coarctation [19]. In in vitro and in vivo experimental models, miR-30d over-expression exerts cardioprotective effects on cardiac remodeling leading to HF, which are related to its ability to 1. inhibit cardiac fibrosis, 2. reduce myocardial cell apoptosis, and 3. regulate the expression of several genes and signaling pathways involved in HF pathogenesis, for instance, by inhibiting the expression of transforming growth factor beta 1 [20,21,22]. In addition, miR-30d over-expression has been shown to prevent in vitro cardiac hypertrophy by counteracting the pro-apoptotic effect of TNF-α on cultured cardiomyocytes directly acting on the MAP-kinase cascade [20,23]. Furthermore, since chronic inflammation is recognized as a major contributing factor in HF [24], and, in particular, NLRP3 (nucleotide-binding oligomerization domain, leucine-rich repeat-containing proteins 3) inflammasome activation plays a major role in it [25,26], it can be hypothesized that miR-30d may also contribute to these processes through its anti-inflammatory properties [22,27].

mir-30d has been shown to exert significant effects by modulating various biological processes involved in cardiac remodeling, apoptosis, and fibrosis. miR-30 family members, including miR-30d, can, indeed, downregulate the expression of connective tissue growth factor (CTGF), a critical mediator of fibrosis, thereby inhibiting extracellular matrix accumulation and fibrosis in the myocardium [28]. In addition, miR-30d has been found to inhibit cardiomyocyte apoptosis by targeting specific pro-apoptotic genes. For instance, overexpression of miR-30d-5p in a rat model of myocardial infarction led to a decreased ratio of phosphorylated p53 to total p53, indicating reduced apoptotic activity in cardiomyocytes [29]. Finally, miR-30d has been shown to have a significant role in modulating cardiac fibrosis by targeting components involved in transforming growth factor-beta (TGF-β) signaling, thereby modulating fibroblast activation and extracellular matrix production [30].

Moving from these bases, miR30d has also been explored as a potential biomarker in patients with HF. Some studies have described a reduced expression of miR30d in blood and cardiac tissue samples of patients with HF [28,29], which have been associated with worse disease severity [28] and one-year mortality [29]. On the contrary, Melman and co-authors have shown that miR-30d plasma levels are increased in patients with HF and dyssynchrony candidates to cardiac resynchronization therapy (CRT), and that they are associated with response to CRT independent of clinical markers of risk [23].

However, acute settings like ACPE may involve distinct regulatory mechanisms due to the rapid onset and progression of the clinical manifestations of disease. By addressing these gaps, miR-30d could emerge as a biomarker for improving outcomes in ACPE patients and beyond, bridging the translational gap from basic research to clinical practice. Despite the growing body of literature on miR-30d in CHF, there is a paucity of data regarding its role in AHF conditions such as ACPE. Therefore, investigating miR-30d expression and function in these acute care clinical settings could establish it as a valuable biomarker for ACPE diagnosis and re-hospitalization risk prediction, ultimately enhancing patient stratification and outcome.

Taking into account the data available on the role of miR30d in HF, it is clear that further studies are needed to better elucidate the potential applications of miR-30d as a biomarker in clinical practice, especially in the clinical setting of patients with ACPE, where no data are available.

In the present study, we aimed to study the expression levels of miR-30d in patients with ACPE at the time of admission in the ED, and to evaluate its performance as a biomarker for the diagnosis of ACPE and as a predictor of risk of mortality and hospital readmission.

## 2. Results

### 2.1. Characteristics of Control Subjects and ACPE Patients Enrolled in the Study

The study enrolled a total of 24 patients with ACPE and 10 control healthy subjects, whose demographic and clinical characteristics are reported in Table 1.

The demographic characteristics did not differ significantly between the two groups (see Table 1).

ACPE patients exhibited a high prevalence of ischemic heart disease (41.5%) followed by hypertensive cardiopathy (20.8%); together, valvular and valvular-hypertensive cardiopathy accounted for 33.3% of cases, and dilatative cardiopathy for 4.2%.

Over the course of the one-year follow-up, 6 patients (12.5%) died, and 10 experienced a new hospital admission related to HF (7 of them in the first 3 months and 3 more in the following 9 months).

All patients evaluated at the one-month follow-up were already discharged from the hospital and did not report acute symptoms related to HF (i.e., NYHA class I-II) at the point of clinical evaluation.

The circulating plasma levels of miR-30d, quantitatively assessed through qRT-PCR, were determined at the time of enrolment (T0) and at the one-month follow-up (T1). These results are shown in Table 1 along with data on the level of hemoglobin and creatinine and of different markers (i.e., NT-proBNP, troponin T, c-reactive protein, copeptin, and Suppression of Tumorigenicity 2 protein–ST2). All biomarkers were determined in both ACPE patients and control healthy subjects at T0, but, at T1, only in patients.

Echocardiography and LUS data obtained during the initial diagnostic work-up are reported in Table 1. The median value of the ejection fraction in ACPE patients was mildly reduced (40%, range 15–70%), and significantly different from that of healthy subjects (58%, range 53–60, *p* = 0.003).

At LUS evaluation, all ACPE patients showed a median of 8 areas positive for B-lines (IQR 8), while pleural effusions were found in 20 patients.

### 2.2. Circulating Levels of miR-30d in Patients with ACPE

**Figure 1:**
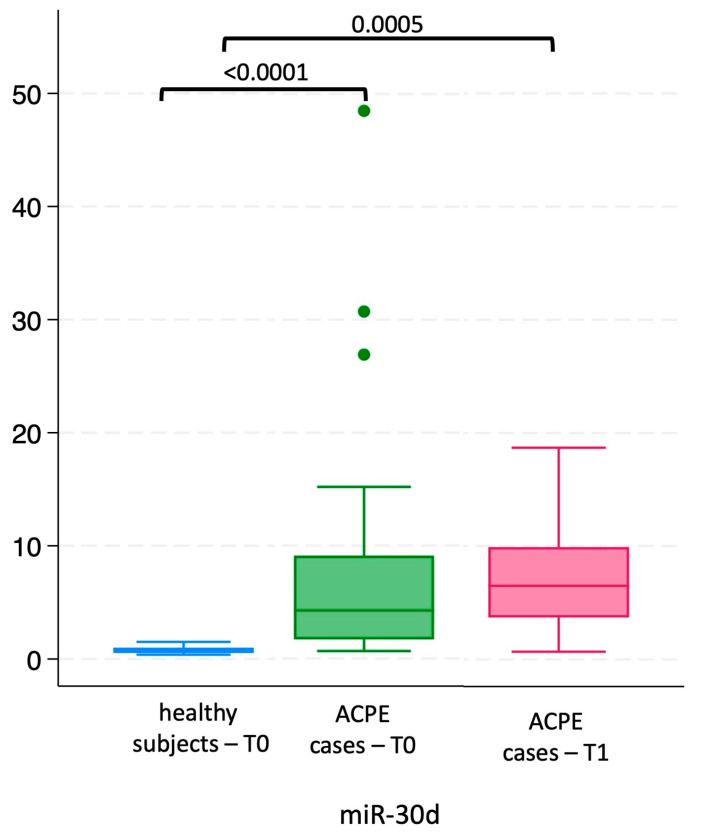
Circulating levels of miR-30d in heathy subjects and ACPE patients at T0 and T1.

Plasma levels of circulating miR-30d were significantly higher in ACPE patients compared to healthy controls, both at enrollment (T0, *p* < 0.01) and at the one-month follow-up (T1, *p* = 0.0005; see Figure 1).

**Figure 2:**
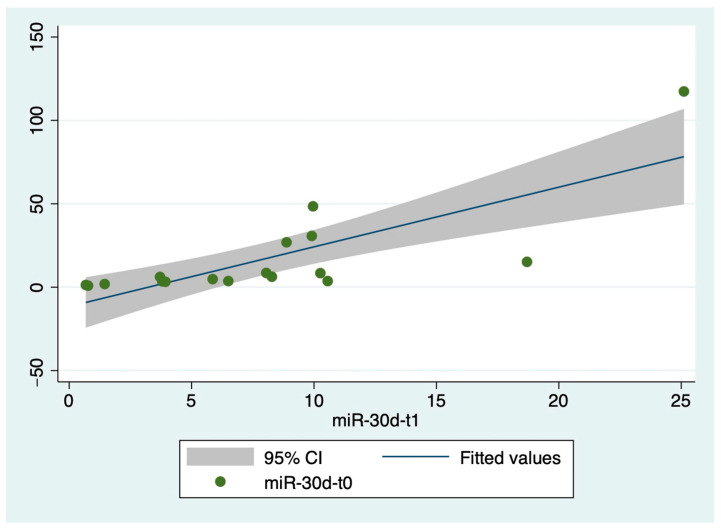
Correlation between circulating levels of miR-30d at T0 and T1.

Although the miR-30d concentration in ACPE patients was almost double at T1 compared to T0, there was no significant difference between miR-30d levels measured at the arrival in the ED and after one-month follow up (Figure 2).

**Figure 3:**
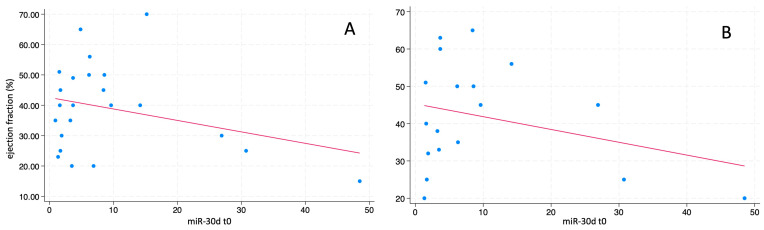
Correlation between the circulating levels of miR-30d at T0 and the ejection fraction at arrival in the ED (panel (**A**)) and at one-year follow up (panel (**B**)) in enrolled ACPE patients.

We investigated the possible correlation between the miR-30d level at T0 and the ejection fraction at ED admission in enrolled ACPE patients. Figure 3 shows the inverse correlation between these factors (correlation coefficient −0.302, Panel A). In addition, we studied the possible correlation between miR-30d level at T0 and the ejection fraction after one-year follow-up in ACPE patients: also, in this case, we found an inverse correlation between miR-30d level at T0 and EF at one-year follow-up (correlation coefficient −0.290, Panel B).

**Figure 4:**
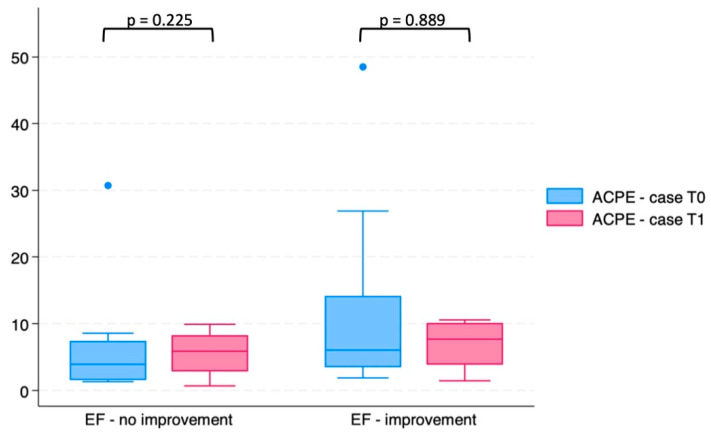
Circulating levels of miR-30d at T0 and T1 in ACPE patients stratified based on EF improvement (>5%) after one-year follow-up.

The median value of the ejection fraction slightly increased in those 18 ACPE patients alive after one-year follow-up (42.5%, range 20–65%; *p* = 0.018 for difference in comparison with the EF at enrollment time—Figure 4).

We then analyzed whether miR-30d levels differed between ACPE patients who showed an improvement in EF (i.e., an increase of at least 5%) at one-year follow-up (10) and those who did not (8). As shown in Figure 3, miR-30d concentrations at T0 and T1 did not differ in these two ACPE patient groups (*p* = 0.07 and *p* = 0.18 at T0 and T1, respectively).

Furthermore, we studied miR-30d concentrations at T0 and T1 in ACPE patients alive at one-year follow-up and those who died during the same period. No significant difference was present (*p* = 0.225 and *p* = 0.889, respectively, for comparison at T0 and T1) in these two groups of patients.

### 2.3. Evaluation of Circulating Levels of miR-30d as Indicators of Risk of Re-Hospitalization in ACPE Patients

**Figure 5:**
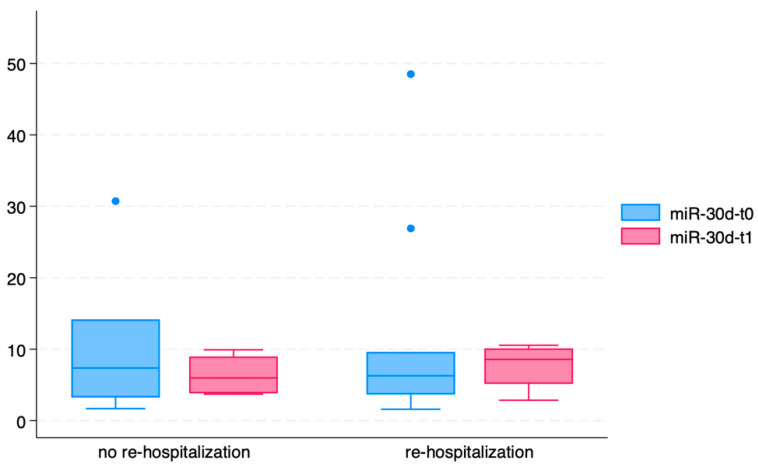
Circulating levels of miR-30d at T0 and T1 in ACPE patients stratified based on the occurrence of re-hospitalization during the one-year follow-up.

We evaluated the circulating levels of miR-30d at T0 and T1 in ACPE patients based on the occurrence of re-hospitalization during the one-year follow-up (Figure 5). miR-30d concentrations were lower, both at T0 (*p* = 0.002) and at T1 (*p* = 0.065), in ACPE patients who needed re-hospitalization in the following year after the index episode.

The ROC curves were used to calculate the diagnostic accuracy of miR-30d in patients with ACPE for the occurrence of re-hospitalization after 3 months or 1 year since the diagnosis.

**Figure 6:**
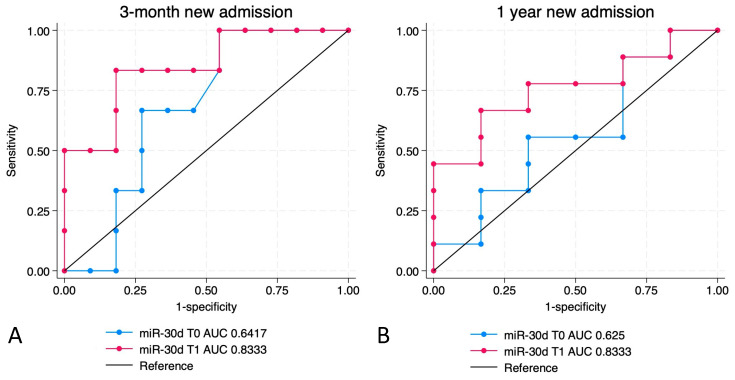
ROC curves for the accuracy of miR-30d levels measured at T0 and T1 for the occurrence of re-hospitalization at 3 months (**A**) and at 1 year (**B**) from the enrollment.

Figure 6, panel A, shows the comparison between the diagnostic accuracy of the concentrations of miR-30d at T0 and T1 for the risk of re-hospitalization at 3 months. The AUC of miR-30d was 0.6417 (95% CI 0.3568–0.9266) and 0.8333 (95% CI 0.6922–1.000) at T0 and T1, respectively (*p* = 0.0032 for comparison between T0 and T1 for re-hospitalization at 3 months).

On the other hand, Figure 6 shows the comparison between the diagnostic accuracy of miR-30d levels at T0 and T1 for the risk of re-hospitalization at 1 year (panel A and B, respectively). The AUC of miR-30d was 0.625 (95% CI 0.2943–0.9557) and 0.8333 (95% CI 0.5495–1.00) at T0 and T1, respectively (*p* = 0.0013 for comparison between T0 and T1 for re-hospitalization at 1 year). Using a ‘cut-off’ value of 3.365 (identified by all used methods, i.e., the Liu, Youden, and nearest point methods), the concentration of miR-30d at T0 showed a sensitivity of 100% and a specificity of 53% in predicting the risk of re-hospitalization at 3 months, and a sensitivity of 90% and a specificity of 45% for the risk of re-hospitalization at 1 year. Using the same test, a cut-off value of 8.165 for the concentrations measured at T1 showed a sensitivity of 83% and a specificity of 82% in predicting the risk of re-hospitalization at 3 months, and a sensitivity of 67% and a specificity of 83% for the risk of re-hospitalization at 1 year.

The levels of miR-30d at T1 show a higher accuracy than the concentration measured at T0 for risk of re-hospitalization both at 3 months and at 1 year.

### 2.4. Evaluation of Circulating Levels of Different Biomarkers as Indicators of Risk of Re-Hospitalization in ACPE Patients

We also measured several circulating biomarkers, some of them already available in clinical practice for ACPE patients: creatinine, troponin T, NT-proBNP, CRP, copeptin, and ST2 (Table 1).

The circulating levels of creatinine, as well as of CRP, were increased in patients with ACPE at T0. However, CRP levels decreased at T1, when the patients had received treatment and were in the compensation phase.

Troponin T and NT-proBNP, considered to be cardiac damage biomarkers, were increased in patients with ACPE, especially at time T0. The levels of copeptin and ST2, which represent more recently introduced biomarkers of HF, were also increased in patients with ACPE, especially at time T0. The levels of these biomarkers decrease at T1 when the activation of the inflammatory cascade, present in the acute phase, resolves after treatment.

**Figure 7:**
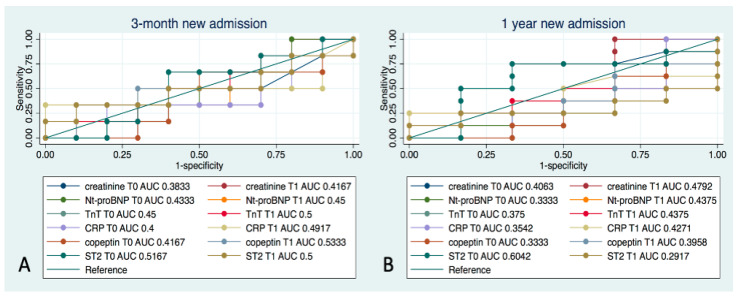
ROC curves for the accuracy of all biomarkers examined (i.e., creatinine, troponin T, NT-proBNP, CRP, copeptin, and ST2) measured at T0 and T1 for the occurrence of re-hospitalization at 3 months (**A**) and at 1 year (**B**) from enrollment.

Figure 7 shows the ROC curves related to the biomarkers examined. Only NT-proBNP and troponin-T reached a high level of sensitivity in predicting the risk of re-hospitalization at both 3 months and 1 year (Figure 6, panel A and B), but with a lower specificity than miR-30d. ST2 at T0 is the only biomarker that shows a moderate accuracy for the risk of re-hospitalization at 1 year (AUC 60.4%). All other biomarker levels did not reach a significant accuracy at either T0 or at T1.

Although this study reports the temporal changes in laboratory biochemical markers and their diagnostic potential, it does not establish a direct correlation between these markers and miR-30d expression levels. Future studies with larger cohorts are needed to explore these potential interactions and their pathophysiological relevance.

## 3. Discussion

HF is still an emerging disease characterized by a severe prognosis and a high mortality, especially for people over the age of 65 years [1,6,7].

We focused our study on patients presenting to the ED with ACPE, one of the most critical clinical pictures of HF.

ACPE is an acute presentation of HF associated with a dramatic increase in respiratory workload that requires rapid recognition due to the risk of immediate deterioration of the patient’s condition [1]. The onset of HF with ACPE represents a negative prognostic factor for disease progression [1]. Advancements in understanding the pathophysiology of HF have led to the discovery of several biomarkers with diagnostic and prognostic potential. However, these biomarkers are still insufficient for accurately stratifying patients based on their risk of adverse clinical outcomes [31,32].

miRs are non-coding single RNAs of approximately 22 nucleotides, which regulate several intracellular processes and up to 50% of coding-genes in a cell [8,9,12]. They can stably exist in various body fluids and have been demonstrated to play roles in intercellular cross talk, with regulatory activity on gene expression and cell-to-cell interactions, influencing apoptosis, proliferation, inflammation, differentiation, and stress response [8,9,12]. Finally, some circulating miRs have been successfully revealed as biomarkers for different conditions such as cancer, brain and liver injury, and cardiovascular diseases, including myocarditis and HF [10,11,33,34,35].

In particular, in the context of HF, miRs have been recently shown to have a role in the modulation of cardiac adaptive processes implied in HF pathogenesis and progression, playing a regulative role between epigenetic, molecular, and cellular adaptive pathways, and cardiovascular outcomes (EF and NYHA class amelioration, reduction in re-hospitalizations due to HF) [36,37].

As already detailed in the Introduction, miR-30d has emerged as one of the most promising among the many miRs investigated in this context. Although it seems to exert a cardioprotective effect in in vitro and in vivo experimental models, its role as a biomarker is controversial. Some studies have described a reduced expression of miR-30d in blood and cardiac tissue samples of patients with HF, which have been associated with worse disease severity and 1-year mortality [15,38]. On the contrary, Melman and co-authors have shown that miR-30d plasma levels are increased in patients with HF and dyssynchrony candidates to cardiac resynchronization therapy (CRT), and that they are associated with response to CRT independent of clinical markers of risk [23]. Therefore, more research is needed to fully elucidate the potential of miR-30d as a biomarker in HF and its potential clinical utility.

The purpose of our study was to evaluate the circulating levels of miR-30d in patients who presented to the ED with ACPE. These patients were evaluated at the time of ED presentation (identified as T0), and at one-month follow-up (T1), and miR-30d expression levels were compared with those of healthy subjects.

We found that the levels of miR-30d were already increased in patients with ACPE, compared to those measured in healthy controls, at the time of first evaluation in the ED (see Figure 1 and Figure 2).

Moreover, the circulating levels of miR-30d were even increased (approximately doubled) in ACPE patients at one-month follow-up, although they did not reach statistical significance (see Figure 1 and Figure 2), when ACPE was clinically resolved. This situation may be attributable to the persistence of the pathophysiologic events underlying the heart disease that led to the acute event represented by ACPE. Therefore, our findings suggest that miR-30d may represent a marker of persistently active disease in patients who have experienced acute HF.

When we studied the possible correlation between miR-30d levels and EF, we found an inverse correlation with the EF measured at the time of ED first evaluation both at T0 and at T1 (see Figure 2). These results were confirmed when we analyzed the possible correlation between miR-30d levels and EF measured at one-year follow-up (see Figure 3, panel B). However, when we stratified ACPE patients based on EF improvement at one-year follow-up, we did not find any difference in miR-30d concentrations measured at T0 and T1 in these two groups of patients (see Figure 4). These data suggest that the increase in miR-30d levels might somehow be related to the reduction in myocardial contractility, but the small number of patients enrolled in our study do not allow us to draw a definitive conclusion on this point.

Analogously, when we stratified ACPE patients based on their vital status (dead/alive) at one year, we did not find any difference in miR-30d levels at both T0 and T1.

These findings indicate that miR-30d levels measured at ED admission and one month later in ACPE patients do not predict therapy response, EF improvement at one year, or one-year mortality. Instead, they appear to reflect acute pathophysiological conditions rather than long-term outcomes.

On the contrary, when we evaluated the accuracy of miR-30d levels in assessing the risk of re-hospitalization in ACPE patients, we found that they showed a robust sensitivity of 100% and 90%, and a specificity level of 53% and 45% in assessing the risk of re-hospitalization at 3 months and 1 year, respectively (see Figure 6). This diagnostic accuracy was significantly higher for miR-30d levels at T1 than at T0, suggesting that the persistence of elevated miR-30d blood levels might be associated with the persistence of those pathogenic mechanisms underlying an increased re-hospitalization risk.

We also compared the ability of miR-30d in the risk stratification of ACPE patients with that of established biomarkers already widely employed in patients with HF, such as NT-proBNP, troponin-T, CRP, copeptin, and ST2. These markers exhibited mild degrees of sensitivity and specificity, and none surpassed those of miR-30d. Our results confirm those already discussed on the potential utility of miR-30d as a biomarker in patients with HF, suggesting that miR-30d could offer additional, valuable information in patient management [23].

These findings suggest that elevated circulating miR-30d levels could serve as an actionable biomarker to identify patients at high risk of re-hospitalization after acute cardiogenic pulmonary edema (ACPE). During clinical practice, monitoring miR-30d could allow healthcare providers to implement targeted follow-up and management protocols, such as more frequent check-ups, enforced patient education, and early intervention strategies, for patients with elevated miR-30d levels. This approach could potentially reduce hospital readmission rates and improve long-term outcomes by enabling the pro-active management of patients at higher risk. Further research could evaluate the cost-effectiveness and clinical benefits of incorporating miR-30d screening in routine post-discharge protocols, potentially guiding healthcare protocols in heart failure management.

In order to better define miR-30d prognostic significance in ACPE patients, it would have been necessary to further expand the case series that, in our study, was limited to 24 ACPE patients. This, along with the absence of a sample size calculation in our proof-of-concept study, represents a major limitation of our study, which needs to be overcome in larger and multicenter studies.

We are aware of the apparent contradiction between the reported protective effects of miR-30d in previous experimental and clinical studies and the increase in miR-30d levels we observed in ACPE patients in our study. A possible explanation may reside in the timing of sample collection, which, in our study, was performed in the ED at the time of first medical evaluation. Indeed, miR-30d might play different role in the context of ACPE compared to CHF. The acute events occurring during ACPE, with its rapid onset and potential for immediate deterioration, could trigger molecular responses distinct from those highlighted in more chronic and stable conditions evaluated in prior studies. Furthermore, miR-30d might have different roles depending on the heart failure stage and specific pathophysiological context. While miR-30d downregulation has been linked to CHF progression, its upregulation during acute episodes might represent a rapid, protective response to acute stress or injury, which aligns with our findings of increased miR-30d during acute exacerbations, as seen in ACPE patients.

While our study helps to clarify the association between elevated miR-30d levels and the risk of rehospitalization in ACPE patients, future research should focus on exploring the temporal dynamics of miR-30d expression across different stages of HF, taking into account the multifaceted nature of this complex syndrome.

Investigating the molecular pathways through which miR-30d influences re-hospitalization rates could provide valuable insights into HF pathophysiology and the regulatory role of miRs in HF progression. Future studies could focus on pathways such as cardiac remodeling, inflammation, and apoptosis, where miR-30d is known to play a role, particularly through mechanisms like inhibition of cardiac fibrosis and reduction of myocardial cell apoptosis [20,21,22,23].

Furthermore, exploring miR-30d as a therapeutic target could significantly advance treatment for HF patients. Potential interventions might involve miR-30d inhibitors or mimics, depending on the regulatory patterns involved in chronic versus acute HF. This aligns with recent research on miR-based treatments in heart diseases, which have shown promising results for HF management. miR-30d regulates pathways critical to HF pathogenesis, including inhibition of fibrosis via suppression of CTGF [28] and TGF-β signaling [20,30], suppression of pro-apoptotic pathways [29], and modulation of inflammatory responses directly targeting the expression of pro-inflammatory cytokines and mediators [39]. These mechanisms highlight miR-30d’s potential as a therapeutic target in HF. Emerging interventional strategies, such as, for example, miR-30d mimics for chronic HF or inhibitors for acute HF, align with these mechanistic insights [40]. Advances in miRNA delivery systems, both viral and non-viral, such as liposome and nanoparticle-based carriers, offer promising avenues to translate these findings into clinical applications by enabling efficient and tissue-specific delivery of miRNA-based therapies [40,41].

The development of miR-30d-based diagnostic assays could facilitate the early identification of patients at high risk of readmission for HF. Using miR-30d as a biomarker for patient stratification and personalized intervention strategies may improve outcomes, as miR-30d demonstrated superior diagnostic accuracy for predicting readmission in this study, outperforming conventional biomarkers in both sensitivity and specificity.

Further research into the long-term effects of elevated miR-30d levels on HF progression and mortality will provide a more comprehensive understanding of its role in chronic disease management. Such studies could help determine whether sustained miR-30d elevation correlates with an increased incidence of complications and poorer functional recovery following acute events.

## 4. Materials and Methods

### 4.1. Patients

We enrolled 24 patients with ACPE evaluated at the ED of the “Città della Salute e della Scienza di Torino—Molinette site” University Hospital, Turin, Italy. Moreover, a group of 10 healthy volunteers was enrolled as controls.

All adult patients presenting at the ED for ACPE (as defined by the European Society of Cardiology guidelines after the standard clinical evaluation) were considered eligible for the study.

Exclusion criteria were: (i) age <18 years; (ii) inability to grant consent to participate in the study; (iii) acute presentation in the form of HF in the absence of ACPE.

**Figure 8:**
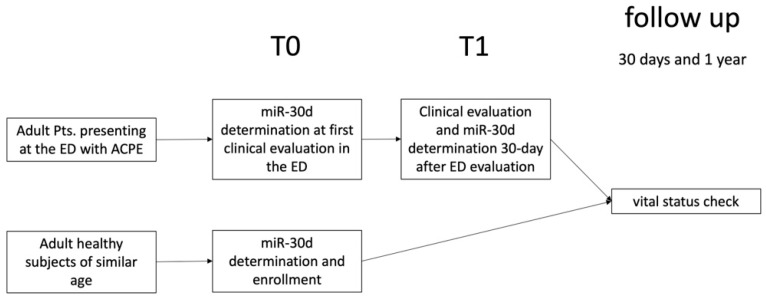
Management of heathy subjects and ACPE patients enrolled in the study.

One month after enrolment, all patients were contacted by telephone in order to offer and arrange a follow-up assessment, including clinical evaluation performed by one of the investigators (G.B. and E.P.) and blood test analysis (Figure 8).

Written informed consent was obtained from all participants, and their samples were anonymized and securely stored for analysis.

The study was approved by the Ethics Committee of our Hospital (“Comitato Etico Interaziendale Azienda Ospedaliero Universitaria Città della Salute e della Scienza di Torino—Azienda Ospedaliera Ordine Mauriziano—Azienda Sanitaria Locale Città di Torino”; n. CS2/139) and conducted in accordance with the ethical standards of the Declaration of Helsinki and its later amendments.

### 4.2. Blood Collection and Plasma Preparation

Blood samples were obtained by clean venipuncture using a 21-gauge infusion set in EDTA-coated tubes at the ED admission (T0) and one-month follow-up (T1). Plasma was recovered by centrifugation at 1600× *g* for 10 min at 4 °C within 2 h after blood collection and immediately stored at −80 °C until analysis.

### 4.3. miR Extraction from Plasma

miRs were isolated from 200 µL of plasma with the miRNeasy Serum/Plasma Advanced Kit (Qiagen, Hilden, Germany) according to the manufacturer’s protocol. After the lysis step with RPL buffer, 3.5 µL of miRNeasy Serum/Plasma spike-in control miRNA-39 from *Caenorhabditis elegans* solution (1.6 × 10^8^ copies/µL) was added as an internal control for RNA extraction efficiency.

Buffer RPP was added to precipitate proteins that are highly concentrated in plasma samples, by centrifugation. Then, the supernatant containing RNA was mixed with 1 volume of isopropanol and applied to the RNeasy UCP MinElute spin column. Total RNA bound to the membrane and the contaminants were washed away using different buffers (RWT, RPE, and 80% ethanol). High-quality RNA, primarily miR and other small RNA, was eluted in 20 µL of RNase-free water and stored at −80 °C.

### 4.4. Reverse Transcription and qRT-PCR

Equal amounts (5 µL) of individual miR extracts were reverse-transcribed using the miScript II RT Kit (Qiagen, Hilden, Germany) according to the manufacturer’s instructions. The kit contains a synthetic miR (miR reverse transcription control, miRTC) that allows to evaluate the reaction efficiency. After cDNA synthesis, the samples were diluted with 20 µL of RNase-free water and 2 µL was then used as a substrate for qRT-PCR. Quantitative PCR was performed using the miScript SYBR Green PCR Kit (Qiagen, Hilden, Germany) with the following miScript Primer Assays (Qiagen, Hilden, Germany), Ce_miR-39_1 (Spike-In Control), Ctrl_miRTC_1, Hs_miR-30d_2 and Hs_miR-16_2. The reactions were run on a QuantStudio 1 Real-Time PCR System (Applied Biosystems, Thermo Fisher Scientific, Waltham, MA, USA) under these cycling conditions: initial denaturation at 95 °C for 15 min, followed by 40 PCR cycles at 94 °C for 15 s, 55 °C for 30 s, and 70 °C for 30 s. The miR-30d expression levels were normalized to those of miR-39 and miR-16 as exogenous and endogenous controls, respectively, and calculated as described by Vandesompele et al. [42].

### 4.5. Biochemical Analysis

Serum creatinine and C-reactive protein (CRP) concentrations were measured by a standardized enzymatic colorimetric method (Creatinine Jaffè Gen.2, Roche Diagnostics GmbH, Mannheim, Germany) and an immune-turbidimetric method (C-Reactive Protein Gen.3, Roche Diagnostics GmbH, Mannheim, Germany), respectively. Plasma concentrations of NT-proBNP and troponin were measured by an electrochemiluminescence method by using Elecsys^®^ NT-proBNP II and Elecsys^®^ Troponin T (Roche Diagnostics GmbH, Mannheim, Germany), respectively. Values were obtained using Roche/Hitachi Cobas c 701 automatic analyzers (Roche Diagnostics GmbH, Mannheim, Germany).

Suppression of Tumorigenicity 2 protein (ST2) concentrations in plasma were measured using the enzyme-linked immunosorbent assay kit Presage^®^ST2, following the manufacturer’s instructions (Critical Diagnostics, San Diego, CA, USA).

Copeptin plasma levels were determined by immunofluorescence using the Copeptin proAVP kit (BRAHMS, Hennigsdorf, Germany—Thermofisher), following the manufacturer’s protocol.

### 4.6. Data Analysis

Descriptive statistics were reported as the median and interquartile range (IQR) for continuous data, or as the number and percentage for ordinal data, as appropriate. The correlation coefficient was used to describe correlation between the ejection fraction (EF) and the miR-30d dosage at the ED admission (T0) and at one-month follow-up (T1).

For each biomarker, the diagnostic accuracy for readmission 3 months and 1 year after the ED evaluation was calculated as sensitivity (i.e., true positive rate in identifying ACPE), specificity (i.e., true negative rate), predictive values (i.e., the probability of ACPE given each biomarker result), likelihood ratios (i.e., the ratio between sensitivity and 1-specificity, and 1-specificity and sensitivity, for the positive and negative likelihood ratio, respectively), and area under the receiver operating characteristic curve (AUC–ROC, the visual plot of sensitivity, on the *y*-axis, and 1-specificity, on the *x*-axis) [43]. We compared the difference in the accuracies by using McNemar’s test for paired data [44].

We used three practical approaches to estimate the optimal cut point for miR30-d: the Liu, Youden, and nearest point methods [45,46].

Based on the intrinsic feature of the study (i.e., proof-of-concept), we planned to enroll a convenient sample of non-consecutive patients presenting to the ED with shortness of breath of cardiac origin and of healthy volunteers, as controls, without sample size calculation.

Data were collected using an Excel spreadsheet (version 16.43; Microsoft, Redmond, WA, USA), and analyses were conducted using Stata (version 17.0/SE; StataCorp, College Station, TX, USA).

## 5. Conclusions

In conclusion, our findings highlight the potential of circulating miR-30d levels as a predictive biomarker for re-hospitalization risk in ACPE patients. Its high sensitivity, alongside its ability to predict the risk of re-hospitalization independently of other biomarkers, holds promise for enhancing the clinician’s ability in patient stratification and subsequent management. Nevertheless, this is a preliminary study with a small sample size, and our findings need further validation in larger, multicenter studies. Moreover, the mechanistic role of miR-30d in the pathophysiology of HF, particularly during ACPE, including its interaction with other mediators and/or biomarkers, requires further investigations. Despite these limitations, our results suggest that circulating miR-30d levels may deserve further investigation as a biomarker that is useful for risk prediction in ACPE patients and may contribute to improving individualized patient care.

## Figures and Tables

**Table 1:** Demographic and clinical characteristics of healthy subjects and ACPE patients enrolled in the study.

	Healthy Subjects (N = 10)	ACPE Patients (N = 24 at T0)	*p*-Value
Age, median (IQR)	78.5 years (9.8)	80.8 years (8.2)	0.465
Gender, Male/Female Ratio	0.66	2	0.151
Underlying Cardiopathy	Hypertensive (20%)	Dilatative 1 (4.2%) Hypertensive 7 (29.3%) Ischemic 10 (41.5%) Valvular 6 (25%)	-
Medications Proton Pump Inhibitors Diuretics Sartans Acetil Salicilic Acid Beta Blockers Statins Calcium Channel Blockers ACE Inhibitors Warfarin Oral Direct Thrombin Inhibitors	7 (70%) 1 (10%) - 5 (50%) 3 (30%) 3 (30%) 2 (20%) 4 (40%) - -	8 (33.3%) 17 (70.8%) 7 (29.2%) 11 (45.8%) 17 (70.8%) 5 (20.8%) 6 (25%) 8 (33.3%) 5 (20.8%) 1 (4.2%)	0.068 0.002 - 1 0.054 0.666 1 0.714 - -
Factors Triggering ACPE	-	ACS ^1^ 6 (25%) Arrhythmias 3 (12.5%) High blood pressure ^2^ 4 (16.6%) Severe anemia 2 (8.4%) COPD exacerbation 1 (4.2%) Infection ^3^ 5 (20.8%) Mechanical cause ^4^ 3 (12.5%)	-
Ejection Fraction (%)	58% (53–60)	40% (15–70)	0.013
miR30d–T0	0.81 (0.4)	3.67 (7.9)	<0.001
miR30d–T1	-	7.28 (6.25)	-
Hemoglobin (g/dL)	13.4 (2.25)	13.2 (4.5)	1
Creatinine–T0 (mg/dL)	0.88 (0.19)	1.63 (1.24)	0.004
Creatinine–T1 (mg/dL)	-	1.27 (0.87)	-
TnT–T0 (ng/L)	5.165 (6)	63.5 (58)	<0.001
TnT–T1 (ng/L)	-	34.41 (36.15)	-
Nt-proBNp–T0 (pg/mL)	154.3 (133.54)	4243.5 (5846)	<0.001
Nt-proBNp–T1 (pg/mL)	-	1791 (1497.4)	-
C-reactive protein–T0 (mg/dL)	2.55 (4.1)	13.25 (30.5)	0.002
C-reactive protein–T1 (mg/dL)	-	2.3 (5.6)	-
Copeptin–T0 (pmol/L)	6.143 (3.01)	141.65 (397.73)	<0.001
Copeptin–T1 (pmol/L)	-	29.32 (23.6)	-
ST2-T0 (ng/mL)	36 (19.56)	68.24 (64.86)	0.003
ST2–T1 (ng/mL)	-	40 (21.38)	-
Median Number of Positive Areas for B-lines (IQR)–T0	-	8 (8)	-
Pleural Effusion (%)–T0	-	5 monolateral (13.5%) 15 bilateral (40.5%)	-

1: acute coronary syndrome; 2: including both uncontrolled hypertension due to therapeutic mistakes or low patient’s compliance and hypertensive crisis; 3: 3 pneumonia, 1 urinary tract infection, and 1 diabetic foot ulcer; 4: including acute mitral regurgitation (2), and acute papillary muscle rupture (1). IQR: interquartile range.

## Data Availability

The data are available upon reasonable request to the corresponding author.
